# Associations of reproductive breast cancer risk factors with expression of stem cell markers in benign breast tissue

**DOI:** 10.3389/fonc.2024.1354094

**Published:** 2024-03-21

**Authors:** Lusine Yaghjyan, Yujing J. Heng, Gabrielle M. Baker, Vanessa C. Bret-Mounet, Divya Murthy, Matt B. Mahoney, Bernard Rosner, Rulla M. Tamimi

**Affiliations:** ^1^ Department of Epidemiology, College of Public Health and Health Professions and College of Medicine, University of Florida, Gainesville, FL, United States; ^2^ Department of Pathology, Beth Israel Deaconess Medical Center, Harvard Medical School, Boston, MA, United States; ^3^ Channing Division of Network Medicine, Department of Medicine, Brigham and Women’s Hospital and Harvard Medical School, Boston, MA, United States; ^4^ Department of Population Health Sciences, Weill Cornell Medicine, New York, NY, United States

**Keywords:** benign breast disease, parity, breastfeeding, age at first child, breast cancer risk

## Abstract

**Background:**

We investigated the associations of reproductive factors known to influence breast cancer risk with the expression of breast stem cell markers CD44, CD24, and ALDH1A1 in benign breast biopsy samples.

**Methods:**

We included 439 cancer-free women with biopsy-confirmed benign breast disease within the Nurses’ Health Study (NHS) and NHSII. The data on reproductive and other breast cancer risk factors were obtained from biennial questionnaires. Immunohistochemistry (IHC) was performed on tissue microarrays. For each core, the IHC expression was assessed using a semi-automated platform and expressed as % of cells that stained positive for a specific marker out of the total cell count. Generalized linear regression was used to examine the associations of reproductive factors with a log-transformed expression of each marker (in epithelium and stroma), adjusted for other breast cancer risk factors.

**Results:**

In multivariate analysis, the time between menarche and age at first birth was inversely associated with CD44 in epithelium (β per 5 years = −0.38, 95% CI −0.69; −0.06). Age at first birth and the time between menarche and age at first birth were inversely associated with ALDH1A1 (stroma: β per 5 years = −0.43, 95% CI −0.76; −0.10 and β = −0.47, 95% CI −0.79; −0.15, respectively; epithelium: β = −0.15, 95% CI −0.30; −0.01 and β = −0.17, 95% CI −0.30; −0.03, respectively). Time since last pregnancy was inversely associated with stromal ALDH1A1 (β per 5 years = −0.55, 95% CI −0.98; −0.11). No associations were found for CD24. The observed associations were similar in premenopausal women. In postmenopausal women, lifetime duration of breastfeeding was inversely associated with stromal ALDH1A1 expression (β for ≥24 *vs.* 0 to <1 months = −2.24, 95% CI 3.96; −0.51, p-trend = 0.01).

**Conclusion:**

Early-life reproductive factors may influence CD44 and ALDH1A1 expression in benign breast tissue.

## 1 Introduction

Parity is a well-established breast cancer risk factor that has been consistently associated with reduced risk of benign breast disease (1), lower breast density (2–5), and reduced breast cancer risk (6–9). Reduction in stem cell pool is currently postulated to be one of the mechanisms underlying the protective effect of pregnancy on breast cancer risk (10). Pregnancy induces a portion of stem cells to differentiate, thus reducing the pool of transformation-susceptible cells in the breast. Stem cells are responsible for subsequent regression of the mammary gland into pre-pregnant state following post-partum involution as well as for regeneration of lactation-component ductal system during subsequent pregnancies (10). It was also suggested that stem cells are at the peak of their abundance and sensitivity to carcinogenic influences in the young mammary gland and during puberty, which is supported by epidemiologic evidence of the highest absolute risk of breast cancer in women exposed to environmental factors early in life (10).

Experimental evidence suggests that early but not late pregnancy reduces the number of mammary stem cells, which supports the evidence on the protective effect of earlier age at first birth and a shorter interval between age at menarche and first birth on breast cancer risk (10–14). Pregnancy is further believed to downregulate signaling pathways known to play a role in mammary stem cell function (10). Thus, a decrease in stem cell pool size and activity with increasing parity could potentially explain its association with breast cancer and intermediate phenotypes (7, 15). However, the evidence of these associations from epidemiological studies remains extremely limited. To fill this gap, we examined the associations of several reproductive factors (parity, age at first birth, breastfeeding, and duration of the time between menarche and first birth) with the expression of well-established stem cell markers CD44 molecule (CD44), CD24 molecule (CD24), and aldehyde dehydrogenase 1 family member A1 (ALDH1A1) in benign breast biopsy samples using prospective data in cancer-free women from the Nurses’ Health Study (NHS) and Nurses’ Health Study II (NHSII) and semi-automated computational pathology method for assessment of stem cell marker expression.

## 2 Materials and methods

### 2.1 Study population

Our analysis included cancer-free women with biopsy-confirmed benign breast disease (BBD) in the NHS and Nurses’ Health Study II (NHSII) cohorts who were previously included in a nested case–control study of breast cancer (16, 17). These prospective cohorts were registered nurses in the United States who were 30–55 years old (NHS) or 25–42 years old (NHSII) at enrollment. After administration of the initial questionnaire, the information on breast cancer risk factors [body mass index (BMI), reproductive history, postmenopausal hormone (PMH) use, and alcohol use] and any diagnoses of cancer or other diseases (including BBD) was updated through biennial questionnaires, which were then confirmed via medical record review (4, 18). Details of this nested case–control study and the BBD assessment have been previously described (16, 17).

Early NHS questionnaires (1976, 1978, and 1980) asked whether the participant had ever been diagnosed with “fibrocystic disease” or “other BBD” and whether she had been hospitalized in relation to this diagnosis. Beginning in 1982, the NHS questionnaires specifically asked about a history of biopsy-confirmed BBD (fibrocystic disease or other BBD). The initial 1989 NHS II questionnaire and all subsequent biennial questionnaires also asked participants to report any diagnosis of BBD and to indicate whether it was confirmed by biopsy or aspiration.

Cases were women with biopsy-confirmed BBD who reported a diagnosis of breast cancer during 1976–1998 for the NHS and 1989–1999 for the NHSII following their BBD diagnosis. Using incidence density sampling, four women with biopsy-confirmed BBD who were free of breast cancer at the time of the matching case’s diagnosis (controls) were matched to the respective case on the year of birth and year of benign breast biopsy (19). We attempted to obtain BBD pathology records and archived biopsy specimens for all cases and controls from their hospital pathology departments; our ability to obtain biopsy blocks did not significantly differ by case and control status. Women were excluded if they had evidence of *in situ* or invasive carcinoma or unknown lesion type at the time of benign breast biopsy (n = 34). All cases and controls from this nested case–control were cancer-free at the time of BBD diagnosis, with an average time of 9 years between biopsy and breast cancer diagnosis date. In the current analysis, we included 439 women who had complete data on reproductive factors and staining results for stem cell markers [92% of all women who had BBD samples (n = 476) used to construct tissue microarrays as described under tissue microarray (TMA) construction]. Women with and without BBD samples were similar with respect to the distribution of breast cancer risk factors (20).

The study protocol was approved by the institutional review boards of the Brigham and Women’s Hospital and Harvard T.H. Chan School of Public Health, and those of participating registries as required. Consent was obtained or implied by return of questionnaires.

### 2.2 Reproductive variables

The data on age at menarche, parity, age at first birth, and breastfeeding were available from baseline and biennial questionnaires, completed closest to the date of the biopsy. Among all eligible women with stem cell marker data (n = 450), the completeness of the data on parity was 97.6% (n = 439). Among parous women with marker readings (n = 398), information on age at first birth and breastfeeding was available for 91.5% and 97.2% of the sample, respectively. Age at menarche was available for 99.3% of the sample. For three women with missing age at menarche, a median value in the study sample was imputed, as performed in previous studies (3, 21, 22).

Age at first birth was modeled both as a categorical variable (<25 years, 25–29 years, and ≥30 years) and as a continuous variable. Parity was defined both as a binary variable (nulliparous and parous) and as categorical variable (1, 2, 3, and ≥4 children). Additionally, the number of children among parous women was modeled as a continuous variable. The lifetime duration of breastfeeding (sum of breastfeeding duration across all births) was classified as none to <1 month, 1 to <12 months, 12 to <24 months, and ≥24 months. Age at menarche was modeled both as a categorical variable (<12 years, 12 years, 13 years, and >13 years) and as a continuous variable. The time interval between menarche and first birth was modeled as continuous variable.

### 2.3 Benign breast biopsy confirmation and BBD subtypes

Hematoxylin and eosin (H&E) breast tissue slides were retrieved for biopsy-confirmed BBD patients who gave permission to review their biopsy records. The slides were previously independently reviewed by one of three pathologists in a blinded fashion; i.e., the evaluating pathologists were blinded to the type of BBD noted on the original diagnosis (23, 24). Any slide identified as having either questionable atypia or atypia was jointly reviewed by two pathologists (23, 24). For each set of slides, a detailed worksheet was completed, and the benign breast biopsy was classified according to the categories of Page et al. (25) as non-proliferative, proliferative without atypia, or atypical hyperplasia (ductal or lobular hyperplasia) (17).

### 2.4 Tissue microarray construction of BBD samples

After a centralized review of H&E-stained slides, we collected archived formalin-fixed paraffin-embedded (FFPE) benign breast biopsy blocks of participants. H&E sections of the corresponding FFPE tissue blocks were re-reviewed by a single pathologist to identify areas of benign proliferative lesions and normal terminal duct lobular units (TDLUs) and to identify the areas from which the cores for the TMAs would be taken. Normal TDLUs were regions of histopathologically normal tissue that may or may not be adjacent to benign lesions (e.g., atypical ductal hyperplasia and usual ductal hyperplasia) (17). TMAs were constructed at the Dana Farber/Harvard Cancer Center (DF/HCC) Tissue Microarray Core Facility by obtaining 0.6-mm cores from benign lesions and TDLUs. For each woman, up to three cores of normal TDLU were included in the TMA blocks. We previously evaluated our TMA construction methods and confirmed a high success rate (76%) of capturing normal TDLUs in these TMA blocks (26).

### 2.5 Immunohistochemistry for stem cell markers

The expression of the stem cell markers was evaluated using an automated immunohistochemistry (IHC) technique that allows the quantification of markers’ expression levels and localization of the target signal to specific cells/structures. For each of the three markers, one 5-μm paraffin section was cut from a single TMA block and then stained with antibodies for CD44, CD24, and ALDH1A1 at the University of Florida Pathology Core Lab on DAKO AutostainerPlus according to the previously standardized protocol with commercial antibodies [DAKO AutostainerPlus, CD44 (DAKO, Glostrup, Denmark) 1:25 dilution; CD24 (Invitrogen, Carlsbad, CA, USA) 1:200 dilution, and ALDH1A1 (Abcam, Cambridge, UK) 1:300 dilution]. Details of this protocol have been described previously (27–29). Briefly, slides were de-paraffinized with xylene and re-hydrated through decreasing concentrations of ethanol to water, including an intermediate step to quench endogenous peroxidase activity (3% hydrogen peroxide in methanol) and transferred to 1× Tris-buffered saline–Tween (TBS-T). For heat-induced antigen retrieval, sections were heated in a steamer while submerged in Citra (BioGenex, Fremont, CA, USA) or Trilogy (Cell Marque, Rocklin, CA, USA) for 30 minutes. Next, slides were 1) rinsed in 1× TBS-T and incubated with a universal protein blocker Sniper (Biocare Medical, Walnut Creek, CA, USA) for 10 minutes (for CD44 and ALDH1A1) or 15 minutes (for CD24); 2) rinsed in 1× TBS-T and co-incubated in primary antibody ALDH1A1, CD24, or CD44 for 1 hour; and 3) rinsed in 1× TBS-T followed by application of conjugated secondary antibody [Mach 2 goat anti-rabbit horse (or mouse) radish peroxidase-conjugated, Biocare Medical, Walnut Creek, CA, USA] for 30 minutes. Detection of antibodies was achieved by incubating slides in 3′3’-diaminobenzidine (Vector Laboratories Inc., Burlingame, CA, USA) for 4 minutes. Slides were counterstained with hematoxylin (Biocare Medical, Walnut Creek, CA) 1:10 for 3 minutes and mounted with Cytoseal XYL (Richard-Allan Scientific, Kalamazoo, MI, USA). The laboratory implemented standard quality control procedures.

### 2.6 Image analysis

Immunoreactivity was quantified using a semi-automated image analysis system, Definiens Tissue Studio software (Munich, Germany), which quantifies tissue marker expression within the context of tissue architecture. For each core, the extent of each marker expression was assessed on a continuous scale as the percent of cells that stain positively (across all intensities) for a specific marker out of the total cell count, separately for epithelium and stroma. Briefly, TMA slides were digitalized at 20× into whole slide images using the Pannoramic Scan 150P (3DHistech, Budapest, Hungary). For each marker, the images were imported into Definiens, and an experienced operator randomly selected a representative TMA as the training TMA (30, 31). On the training TMA, the operator selected 12 training cores that were assessed as >0 to <1 (n = 3), 1 to 10 (n = 3), >10 to 50 (n = 3), and >50% (n = 3) by the pathologist to optimize a Definiens algorithm for automated IHC assessment. Definiens only allows a maximum of 12 cores for algorithm training. The minimum positive IHC staining threshold in Definiens was set using the pathologist’s manual reads as a reference. The optimized Definiens algorithm segmented each tissue core into epithelium, stroma, fat, and background; detected the number of cells; and quantitated the IHC stains. The current analysis was specifically focused on the expression of stem cell markers in normal TDLU cores for the following reasons: 1) we specifically targeted normal TDLUs in the construction of these TMAs within NHS/NHSII, and thus, the number of women with benign lesion cores was smaller and would not allow to draw meaningful conclusions; 2) in our earlier reliability study, we observed higher heterogeneity within benign lesion cores, as they were represented by various lesion types (29); and 3) we were interested in the underlying changes in the breast tissue happening early in the process of breast carcinogenesis, and thus, normal TDLUs were more relevant to address our research questions. Staining results for stroma were available for 423, 434, and 408 women for CD44, CD24, and ALDH1A1, respectively, and the staining results for epithelium were available for 396, 409, and 393 women, respectively.

### 2.7 Covariate information

Information on breast cancer risk factors was obtained from the biennial questionnaires closest to the date of the biopsy. Women were considered to be postmenopausal if they reported 1) no menstrual periods within the 12 months before biopsy with natural menopause, 2) bilateral oophorectomy, or 3) hysterectomy with one or both ovaries retained and were 54 years or older for ever-smokers or 56 years or older for never smokers (32, 33).

### 2.8 Statistical analysis

We modeled marker expression (weighted average across available cores for a woman) both as log-transformed to improve normality of residuals and as dichotomous using 10% cut-offs based on the results of our prior reliability study and distribution in our sample (29).

First, we used multivariate linear regression to examine the associations of parity, age at menarche, age at first birth, breastfeeding, time since last birth and interval between menarche and first birth with continuous expression of each of the markers, adjusted for the following covariates: age (continuous), BMI (continuous), a family history of breast cancer (yes/no), menopausal status/postmenopausal hormone use (premenopausal, postmenopausal/no hormones, postmenopausal/past hormones, postmenopausal/current hormones, and postmenopausal/unknown hormone use status), NHS cohort (NHSI and NHSII), benign breast disease subtype (non-proliferative, proliferative without atypia, and proliferative with atypia), and alcohol use (none, >0 to <5 g/day, and ≥5 g/day). Additionally, in the analysis of the association of breastfeeding, the estimates were adjusted for parity and age at first birth. In the analysis of the associations of parity and age at first birth, the risk estimates were mutually adjusted for these two variables. In the analysis of the interval between menarche and first birth, the estimates were adjusted for parity.

The analyses of all reproductive variables except nulliparity and age at menarche were limited to parous women only. Parity, age at first birth, and age at menarche were modeled as both continuous and categorical, and breastfeeding was modeled as categorical. The lowest category for parity (one child), age at first birth (<25 years), and breastfeeding (0 to <1 month) were used as the reference. To assess the overall trend for each of the categorical reproductive variables, we used respective medians within each category. The duration of the interval between menarche and first birth and the time since the last birth were modeled as continuous variables. Next, we used logistic regression to examine the associations of reproductive factors with dichotomized marker expression while using the same adjustment approaches. Next, among parous women, we also examined the associations in mutually adjusted models that included all reproductive factors except the time between age at menarche and first birth. Finally, in exploratory analyses, we also examined the associations by menopausal status. The results of this stratified analysis, however, should be interpreted with caution due to the small number of observations in some strata. The analyses were performed using SAS software (version 9.4, SAS Institute, Cary, NC, USA).

## 3 Results

Among 439 cancer-free women in this study, 128 (29.2%) had non-proliferative disease, 241 (54.9%) had proliferative disease without atypia, and 70 (16.0%) had atypical hyperplasia, similar to the previously reported distributions (24). The average age at the biopsy was 45 years (range 17–67 years). The sample included predominantly premenopausal women at the biopsy (68.6%). The majority of women were parous (90.7%), and of those, the majority had at least two children (89.9%) and breastfed for at least 1 month (63.1%). The average age at first birth was 24.9 years (range 15–39 years). **Table 1** presents the age-adjusted characteristics of women in the study by nulliparous status. The distribution of markers’ expression by BBD subtype is presented in **Supplementary Table 1**.

**Table 1:** Age-adjusted characteristics of women at the time of the biopsy, stratified by parous status.

Characteristic	Parous n = 398	Nulliparous n = 41
Mean (SD)		
**Age (years)** ^a^	45.75 (8.95)	42.85 (11.91)
** Age at menopause (years)**	48.51 (4.65)	46.09 (5.36)
** Body mass index (kg/m^2^)**	23.87 (4.07)	24.16 (4.27)
** Alcohol use (g/day)**	5.24 (8.43)	3.30 (3.84)
** Parity**	3.05 (1.39)	NA
** Age at first birth (years)**	24.85 (3.47)	NA
** Age at menarche (years)**	12.55 (1.37)	12.56 (1.26)
** CD44 normal TDLU epithelium %**	39.77 (35.87)	40.91 (32.76)
** CD44 normal TDLU stroma %**	17.96 (27.32)	24.67 (28.5)
** CD24 normal TDLU epithelium %**	28.74 (22.58)	35.92 (23.06)
** CD24 normal TDLU stroma %**	8.68 (13.77)	8.79 (11.94)
** ALDH1A1 normal TDLU epithelium %**	26.98 (19.6)	30.68 (13.89)
** ALDH1A1 normal TDLU stroma %**	11.82 (13.97)	10.23 (7.17)
Percentages		
Breastfeeding		
** 0** to <**1 month**	36	NA
** 1** to <**12 months**	32	NA
** 12** to <**24 months**	19	NA
** ≥24 months**	14	NA
Family history of breast cancer	13	16
Smoking status		
** Never smoked**	46	39
** Past smoker**	35	37
** Current smoker**	19	24
Menopausal status/postmenopausal hormone use		
** Premenopausal**	69	72
** Never used**	9	5
** Past use**	4	1
** Current use** ** Unknown**	6 12	9 13
Benign breast disease subtypes		
** Non-proliferative**	30	29
** Proliferative without atypia**	54	64
** Proliferative with atypia**	16	6

Values are means (SD) and percentages and are standardized to the age distribution of the study population.

SD, standard deviation; NA, not applicable; TDLU, terminal duct lobular unit.

a Value is not age adjusted.

Age and BMI-adjusted associations of reproductive factors with stem cell markers are presented in **Supplementary Table 2**. In multivariate analysis (**Table 2**), nulliparous status and, among parous women, number of children, age at first birth, and duration of breastfeeding were not associated with CD44 expression in epithelium or stroma. Younger age at menarche was marginally associated with lower CD44 expression in stroma and epithelium (p-trend = 0.12 and 0.08, respectively), with significant estimates found for age at menarche 12 *vs.* >13 years (β = −1.56, 95% CI −2.82; −0.31 and β = −0.91, 95% CI −1.58; −0.25, respectively). The time between menarche and age at first birth was inversely associated with CD44 in epithelium (β per 5-year increase = −0.38, 95% CI −0.69; −0.06). Age at first birth and the time between menarche and age at first birth were inversely associated with ALDH1A1 (stroma: β per 5-year increase = −0.43, 95% CI −0.76; −0.10 and β = −0.47, 95% CI −0.79; −0.15, respectively; epithelium: β = −0.15, 95% CI −0.30; −0.01 and β = −0.17, 95% CI −0.30; −0.03, respectively). Associations of the time between menarche and age at first birth with CD44 and ALDH1A1 were similar when the models were additionally adjusted for age at menarche (data not shown). The time since the last pregnancy was inversely associated with stromal ALDH1A1 (β per 5 years = −0.55, 95% CI −0.98; −0.11). We found no associations of any of the reproductive factors with continuous CD24 expression. Finally, associations of age at menarche with CD44 expression in stroma and epithelium became more pronounced when the models were mutually adjusted for all reproductive factors among parous women (p-trend = 0.01 for both stroma and epithelium) (**Supplementary Table 3**). Associations of age at first birth with CD44 in epithelium and ALDH1A1 in stroma as well as associations of age at menarche with ALDH1A1 in epithelium were similar in mutually adjusted models (**Supplementary Table 3**).

**Table 2:** Associations of reproductive factors with log-transformed expression of stem cell markers in benign breast biopsy samples (β coefficients and 95% confidence intervals).

Reproductive factor	CD44				CD24				ALDH1A1			
	N	In epithelium	N	In stroma	N	In epithelium	N	In stroma	N	In epithelium	N	In stroma
**Nulliparity****^a^** **Nulliparous** **Parous**	28 335	0.41 (−0.43; 1.26) Reference	34 354	0.21 (−1.33; 1.74) Reference	34 342	0.17 (−0.31; 0.64) Reference	36 362	−0.54 (−1.54; 0.46) Reference	29 336	0.22 (−0.13; 0.56) Reference	30 345	0.59 (−0.20; 1.39) Reference
**Breastfeeding, months****^b^** **0** to <**1** **1** to <**12** **12** to <**24** **≥24** **p-Trend**	110 105 63 45 323	Reference 0.18 (−0.37; 0.73) −0.33 (−1.00; 0.34) 0.03 (−0.72; 0.78) 0.65	120 112 64 46 342	Reference −0.17 (−1.24 0.90) −0.39 (−1.70; 0.93) −0.38 (−1.87; 1.10) 0.56	113 107 64 46 330	Reference 0.12 (−0.24; 0.48) −0.37 (−0.81; 0.06) −0.10 (−0.59; 0.38) 0.25	124 114 65 47 350	Reference 0.43 (−0.27; 1.13) −0.22 (−1.08; 0.65) −0.26 (−1.24; 0.71) 0.35	110 105 64 46 325	Reference −0.08 (−0.32; 0.16) −0.10 (−0.38; 0.19) −0.04 (−0.36; 0.29) 0.79	116 110 65 42 333	Reference 0.03 (−0.53; 0.59) −0.09 (−0.77; 0.59) −0.01 (−0.81; 0.79) 0.87
**Parity** **^c^** **1** **2** **3** **≥4** **p-Trend**	34 96 105 97 332	Reference 0.28 (−0.55; 1.13) −0.18 (−1.04; 0.69) 0.46 (−0.43; 1.35) 0.45	37 104 111 99 351	Reference −0.15 (−1.79; 1.49) −0.71 (−2.40; 0.98) 0.39 (−1.35; 2.13) 0.54	35 101 102 101 339	Reference 0.15 (−0.39; 0.69) 0.01 (−0.54; 0.57) 0.18 (−0.40; 0.75) 0.72	38 108 110 103 359	Reference −0.28 (−1.35; 0.78) −0.42 (−1.53; 0.68) −0.33 (−1.47; 0.81) 0.65	35 99 104 96 334	Reference 0.14 (−0.23; 0.50) −0.03 (−0.40; 0.35) 0.09 (−0.30; 0.47) 0.94	35 103 106 98 342	Reference 0.08 (−0.79; 0.94) 0.14 (−0.76; 1.04) 0.22 (−0.70; 1.14) 0.58
**Parity continuous** **^c^**	332	0.05 (−0.12; 0.23)	351	0.12 (−0.24; 0.47)	339	0.06 (−0.05; 0.18)	359	0.003 (−0.23; 0.23)	334	−0.05 (−0.12; 0.03)	342	−0.03 (−0.21; 0.15)
**Age at 1st birth** **^d^** **<25** **25–29** **≥30** **p-Trend**	180 123 29 332	Reference −0.37 (−0.86; 0.11) −0.63 (−1.49; 0.23) 0.08	193 128 30 351	Reference −0.70 (−1.65; 0.25) −0.60 (−2.29; 1.10) 0.26	184 125 30 339	Reference 0.20 (−0.11; 0.52) 0.34 (−0.21; 0.89) 0.14	194 134 31 359	Reference 0.13 (−0.48; 0.75) 0.62 (−0.48; 1.73) 0.27	180 124 30 334	Reference −0.08 (−0.29; 0.13) −0.19 (−0.56; 0.18) 0.26	188 124 30 342	Reference −0.62 (−1.11; −0.12) −0.54 (−1.42; 0.33) 0.05
**Age at 1st birth (per 5 years)** **^d^**	332	−0.30 (−0.63; 0.03)	351	−0.35 (−0.99; 0.29)	339	0.19 (−0.02; 0.41)	359	0.26 (−0.16; 0.69)	334	−0.15 (−0.30; −0.01)	342	−0.43 (−0.76; −0.10)
**Age at menarche** **^e^** **<12** **12** **13** **>13** **p-Trend**	88 95 109 71 363	−0.61 (−1.31; 0.09) −0.91 (−1.58; −0.25) −0.61 (−1.25; 0.04) Reference 0.08	91 106 114 77 388	−1.21 (−2.55; 0.12) −1.56 (−2.82; −0.31) −1.48 (−2.71; −0.24) Reference 0.12	87 98 114 77 376	0.15 (−0.27; 0.58) −0.17 (−0.57; 0.23) 0.19 (−0.19; 0.58) Reference 1.00	91 108 116 83 398	0.88 (−0.01; 1.76) 0.55 (−0.28; 1.37) 0.53 (−0.28; 1.34) Reference 0.07	83 93 114 75 365	−0.27 (−0.56; 0.03) −0.22 (−0.50; 0.06) −0.10 (−0.37; 0.16) Reference 0.05	86 102 112 75 375	−0.56 (−1.23; 0.11) −0.48 (−1.11; 0.14) −0.76 (−1.37; −0.16) Reference 0.28
**Age at menarche (per 5 years)** **^e^**	363	0.63 (−0.21, 1.47)	388	1.07 (−0.54, 2.69)	376	−0.07 (−0.58; 0.45)	398	−0.91 (−1.98; 0.16)	365	0.24 (−0.11; 0.60)	375	0.43 (−0.38; 1.24)
**Time between menarche and age at 1st birth (per 5 years)** **^f^**	332	−0.38 (−0.69; −0.06)	351	−0.53 (−1.15; 0.09)	339	0.18 (−0.03; 0.38)	359	0.31 (−0.10; 0.72)	334	−0.17 (−0.30; −0.03)	342	−0.47 (−0.79; −0.15)
**Time since last birth (per 5 years)****^b^**	325	−0.22 (−0.62; 0.18)	344	−0.31 (−1.11; 0.50)	332	0.21 (−0.05; 0.48)	352	−0.48 (−1.01; 0.05)	327	−0.12 (−0.31; 0.06)	335	−0.55 (−0.98; −0.11)

BMI, body mass index; NHS, Nurses’ Health Study.

a Adjusted for age (continuous), BMI (continuous), age at menarche (<12, 12, 13, and >13), a family history of breast cancer (yes/no), menopausal status/postmenopausal hormone use (premenopausal, postmenopausal/no hormones, postmenopausal/past hormones, postmenopausal/current hormones, and postmenopausal/unknown hormone use status), NHS cohort (NHSI and NHSII), benign breast disease subtype (non-proliferative, proliferative without atypia, and proliferative with atypia), and alcohol use (none, >0 to <5 g/day, and ≥5 g/day).

b Among parous women only: adjusted for age (continuous), BMI (continuous), age at menarche (<12, 12, 13, and >13), parity, age at first child’s birth, a family history of breast cancer (yes/no), menopausal status/postmenopausal hormone use (premenopausal, postmenopausal/no hormones, postmenopausal/past hormones, postmenopausal/current hormones, and postmenopausal/unknown hormone use status), NHS cohort (NHSI and NHSII), benign breast disease subtype (non-proliferative, proliferative without atypia, and proliferative with atypia), and alcohol use (none, >0 to <5 g/day, and ≥5 g/day).

c Among parous women only: adjusted for age (continuous), BMI (continuous), age at first birth, age at menarche (<12, 12, 13, and >13), a family history of breast cancer (yes/no), menopausal status/postmenopausal hormone use (premenopausal, postmenopausal/no hormones, postmenopausal/past hormones, postmenopausal/current hormones, and postmenopausal/unknown hormone use status), NHS cohort (NHSI and NHSII), benign breast disease subtype (non-proliferative, proliferative without atypia, and proliferative with atypia), and alcohol use (none, >0 to <5 g/day, and ≥5 g/day).

d Among parous women only: adjusted for age (continuous), BMI (continuous), parity, age at menarche (<12, 12, 13, and >13), a family history of breast cancer (yes/no), menopausal status/postmenopausal hormone use (premenopausal, postmenopausal/no hormones, postmenopausal/past hormones, postmenopausal/current hormones, and postmenopausal/unknown hormone use status), NHS cohort (NHSI and NHSII), benign breast disease subtype (non-proliferative, proliferative without atypia, and proliferative with atypia), and alcohol use (none, >0 to <5 g/day, and ≥5 g/day).

e Adjusted for age (continuous), BMI (continuous), parous status (nulliparous and parous), a family history of breast cancer (yes/no), menopausal status/postmenopausal hormone use (premenopausal, postmenopausal/no hormones, postmenopausal/past hormones, postmenopausal/current hormones, and postmenopausal/unknown hormone use status), NHS cohort (NHSI and NHSII), benign breast disease subtype (non-proliferative, proliferative without atypia, and proliferative with atypia), and alcohol use (none, >0 to <5 g/day, and ≥5 g/day).

f Among parous women only: adjusted for age (continuous), BMI (continuous), parity, a family history of breast cancer (yes/no), menopausal status/postmenopausal hormone use (premenopausal, postmenopausal/no hormones, postmenopausal/past hormones, postmenopausal/current hormones, and postmenopausal/unknown hormone use status), NHS cohort (NHSI and NHSII), benign breast disease subtype (non-proliferative, proliferative without atypia, and proliferative with atypia), and alcohol use (none, >0 to <5 g/day, and ≥5 g/day).

When marker expression was modeled as dichotomous, the time since last birth was positively associated with stromal CD24 expression (OR per 5 years = 1.85, 95% CI 1.05; 3.26) and inversely associated with stromal ALDH1A1 expression (OR per 5 years = 0.60, 95% CI 0.38; 0.94) (**Supplementary Table 4**). There was a suggestive trend of an inverse association between longer duration of breastfeeding and dichotomous stromal CD24 expression (p-trend = 0.03) (**Supplementary Table 4**), which, however, did not have a clear pattern.

The associations with CD44 were similar in premenopausal women (**Supplementary Table 5**), while in postmenopausal women, we observed positive associations of age at menarche with CD44 expression in stroma and epithelium (**Supplementary Table 6**). The significant trend for associations for categorical parity with CD44 in stroma and epithelium in postmenopausal women did not have a clear pattern and should be interpreted with caution given small numbers in some strata. For CD24, overall patterns were similar in premenopausal women. Additionally, we observed a positive association of age at first birth with epithelial CD24 expression (β per 5 years = 0.31, 95% CI 0.04; 0.57) and an inverse association of age at menarche with stromal CD24 expression (β per 5 years = −1.43, 95% CI −2.59; −0.27). In postmenopausal women, age at menarche was positively associated with epithelial CD24 expression (β per 5 years = 1.18, 95% CI 0.14; 2.22). Similar to overall results, in premenopausal women, age at first birth and the interval between age at menarche and age at first birth were both inversely associated with stromal (β per 5 years = −0.54, 95% CI −0.95; −0.13 and β = −0.59, 95% CI −0.98; −0.21, respectively) and epithelial (β per 5 years = −0.25, 95% CI −0.41; −0.08 and β = −0.23, 95% CI −0.39; −0.07, respectively) ALDH1A1 expression. In premenopausal women, the time since last birth was inversely associated with stromal ALDH1A1 (β per 5 years = −0.60, 95% CI −1.17; −0.03). These associations were not observed in postmenopausal women, but the time since last birth was inversely associated with epithelial ALDH1A1 expression (β per 5 years = −0.42, 95% CI −0.70; −0.14). In postmenopausal women, lifetime duration of breastfeeding appeared to be inversely associated with stromal expression of ALDH1A1 (β for ≥24 *vs.* 0 to <1 months = −2.24, 95% CI 3.96; −0.51, p-trend = 0.01).

## 4 Discussion

Among 439 cancer-free women in our study, we found inverse associations of age at first birth and the time between menarche and first birth with expression of CD44 and ALDH1A1 and positive, though marginally significant, associations of age at menarche with CD44 expression. The lifetime duration of breastfeeding was inversely associated with stromal ALDH1A1 expression in postmenopausal women. No associations were observed for nulliparous status or number of children.

It has been suggested that breast tissue changes during pregnancy may explain its long-term protective effect on the risk of breast cancer (34). Full-term pregnancy influences breast tissue in a variety of mechanisms, including hormonal signaling changes, alterations in gene methylation and expression, long-term reduction in the circulating hormone levels, and life-long decrease in the number of stem cells (7, 14, 35–39). Further, it was suggested that terminal differentiation of mammary stem cells during breastfeeding reduces the pool of breast stem cells at risk of mutation, which could explain the subsequent long-term reduction in the risk of breast cancer (40). However, the evidence from epidemiologic studies with cancer-free women remains very limited. An earlier study that investigated possible associations between the size of stem/progenitor cell population and parity by examining the percentage of ALDH1A1+ cells in breast epithelium found no significant differences in nulliparous *vs.* parous women (41). In our study, we did not find significant associations of parous status or the number of children with the expression of any of the three markers, though nulliparous women had suggestive evidence of greater expression (with exception of stromal CD24) of these markers. Another investigation utilizing normal adjacent tissue from women with triple-negative breast cancer found that women with a greater presence of stem cells were less likely to have breastfed or had a shorter duration of breastfeeding (42). In our study, breastfeeding duration appeared to be inversely associated with the expression of stromal ALDH1A1 among postmenopausal women. However, these results need further confirmation due to the small number of women in some of the strata. Finally, we observed an inverse association of the time since last birth with the expression of stromal ALDH1A1, overall and in premenopausal women, and an inverse association with epithelial ALDH1A1 in postmenopausal women. Whether this potential decrease in stem cell marker expression could explain the long-term reduction in breast cancer risk in parous women is yet to be determined.

It was suggested that earlier menarche and thus earlier exposure to estrogens may alter stem cell functions in the breast tissue (43). As pregnancy induces cellular differentiation, earlier age at first birth and a shorter period between age at menarche and first birth both reduce breast cancer risk by shortening the period when breast tissue, including stem cells, remains highly susceptible to various potentially carcinogenic insults (13, 44). Contrary to these mechanisms, in our study, we found positive associations of age at menarche with expression of CD44 in stroma and epithelium and inverse associations of age at first birth and the interval between menarche and age at first birth with CD44 and ALDH1A1 expression.

Our study is the first to date to explore associations of several reproductive variables with the expression of breast stem cell markers CD44, CD24, and ALDH1A1 in cancer-free women. We utilized data from the Nurses’ Health Study and Nurses’ Health Study II, established cohorts with more than 30 years of follow-up, with confirmation of benign breast disease status, and comprehensive breast cancer risk factor information. The following few limitations should be noted. Even though the data in these cohorts have been collected prospectively, measurement error for some of the reproductive variables particularly in postmenopausal women cannot be ruled out completely. For example, in the previous studies, the findings on the accuracy of recall for age at menarche have been conflicting (45–47), which could potentially influence our findings for the interval between menarche and first birth. Next, we recognize that biopsy samples come from a specific area of the breast. In our previous studies, we demonstrated that this tissue sampling method still provides strong evidence for formulating *a priori* hypotheses and meaningful findings for involution of breast tissue (48), identification of breast cancer risk markers (17, 49, 50), and associations with various breast cancer risk factors, suggesting only a minimal impact of this limitation on research findings (51). Only cancer-free women with routine clinical biopsy with BBD diagnosis are included in our study; however, since our analysis is focused on normal TDLUs, the findings are likely to be generalizable to all cancer-free women and not necessarily limited only to women with BBD diagnosis. Even though the positivity of the staining was calculated taking into account any staining intensity, the associations were not examined separately for each intensity level. We do not focus specifically on the intensity, as there are accumulating data indicating that staining intensity can be affected by both storage time and variability in processing (52). Because our samples were collected from across the United States over a large period of time, we do not feel that staining intensity would be a reliable measure (17). Finally, the results of the stratified analysis by menopausal status should be interpreted with caution given the small number of postmenopausal women in our sample.

In conclusion, we examined the associations of several reproductive variables with the expression of stem cell markers CD44, CD24, and ALDH1A1 in cancer-free women. Our findings suggest positive associations of age at menarche with CD44 expression and inverse associations of age at first birth and the time between menarche and first birth with expression of CD44 and ALDH1A1 as well as inverse associations of lifetime duration of breastfeeding with stromal ALDH1A1 expression in postmenopausal women. Future studies are needed to confirm these findings and to explain the potential biological mechanisms.

## Data availability statement

The data that support the findings of this study are available from the Nurses’ Health Studies, however they are not publicly available. Investigators interested in using the data can request access, and feasibility will be discussed at an investigators’ meeting. Limits are not placed on scientific questions or methods, and there is no requirement for co-authorship. Additional data sharing information and policy details can be accessed at http://www.nurseshealthstudy.org/researchers.

## Ethics statement

The studies involving humans were approved by the institutional review boards of the Brigham and Women’s Hospital and Harvard T.H. Chan School of Public Health, and those of participating registries as required. Written informed consent was obtained or implied by return of questionnaires. The studies were conducted in accordance with the local legislation and institutional requirements.

## Author contributions

LY: Conceptualization, Formal analysis, Funding acquisition, Investigation, Methodology, Writing – original draft, Writing – review & editing. YH: Investigation, Methodology, Writing – review & editing. GB: Data curation, Writing – review & editing. VB-M: Investigation, Writing – review & editing. DM: Data curation, Writing – review & editing. MM: Project administration, Resources, Writing – review & editing. BR: Methodology, Writing – review & editing. RT: Conceptualization, Funding acquisition, Investigation, Methodology, Writing – review & editing.
