# Supplementary material for: Associations of reproductive breast cancer risk factors with expression of stem cell markers in benign breast tissue
Source: Front Oncol. 2024 Mar 21;14:1354094. doi: 10.3389/fonc.2024.1354094 (PMC10991780; doi:10.3389/fonc.2024.1354094)
Supplement: Supplementary file 1 [file Table_1.docx]

**Supplementary table 1. Distribution of stem cell markers by BBD subtype (mean, SD, range)**

| Marker **expression** | **Non-proliferative**  **(n=128)** | **Proliferative without atypia**  **(n=241)** | **Proliferative with atypia**  **(n=70)** |
| --- | --- | --- | --- |
| **CD44 epithelium** | 37.8 (37.5  0-100 | 38.5 (36.1)  0-100 | 48.8 (33.3)  0.9-100 |
| **CD44 stroma** | 19.0 (28.9)  0-96.2 | 17.5 (27.6)  0-94.9 | 21.6 (28.3)  0-93.8 |
| **CD24 epithelium** | 28.1 (22.3)  0.5-98.1 | 28.3 (23.1)  0-97.6 | 33.7 (23.4)  0.7-82.5 |
| **CD24 stroma** | 7.0 (13.0)  0-63.5 | 8.4 (13.5)  0-83.6 | 12.6 (15.9)  0-73.0 |
| **ALDH1A1 epithelium** | 28.6 (21.0)  0.5-90.8 | 26.0 (19.5)  0.5-93.4 | 27.7 (16.4)  0.9-61.1 |
| **ALDH1A1 stroma** | 11.3 (13.6)  0-85.0 | 10.3 (11.9)  0-67.2 | 17.9 (18.1)  0-64.6 |

**Supplementary table 2. Age and BMI-adjusted associations of reproductive factors with log-transformed expression of stem cell markers in benign breast biopsy samples (β coefficients and 95% Confidence intervals)**

|  | **CD44** | | **CD24** | | **ALDH1A1** | |
| --- | --- | --- | --- | --- | --- | --- |
| **Reproductive factor** | **Epithelium** | **Stroma** | **Epithelium** | **Stroma** | **Epithelium** | **Stroma** |
| **Nulliparity**  **Nulliparous**  **Parous** | 0.34 (-0.46; 1.14) Reference | 0.18 (-1.28; 1.64)  Reference | 0.24 (-0.20; 0.69)  Reference | -0.32 (-1.27; 0.63)  Reference | 0.21 ( -0.13; 0.55)  Reference | 0.64 ( -0.16;1.43)  Reference |
| **Breastfeeding, months**  **0-<1**  **1-<12**  **12-<24**  **≥24**  **p-trend** | Reference  0.23 (-0.34; 0.80)  -0.21 (-0.86; 0.45)  0.14 (-0.60; 0.88)  0.87 | Reference  0.05 (-1.04; 1.28)  -0.17 (-1.44; 1.10)  0.14 (-1.30; 1.57)  0.97 | Reference  0.11 (-0.24; 0.46)  -0.19 (-0.60; 0.21)  -0.01 (-0.47; 0.45)  0.54 | Reference  0.32 (-0.39;1.03)  0.06 (-0.78; 0.89)  -0.22 (-1.67; 0.72)  0.54 | Reference  -0.08 (-0.32; 0.16)  -0.10 (-0.38; 0.19)  -0.04 (-0.36; 0.29)  0.63 | Reference  0.06 (-0.52; 0.64)  -0.28 ( -0.96; 0.40)  0.12 (-0.67; 0.91)  0.88 |
| **Parity**  **1**  **2**  **3**  **≥4**  **p-trend** | Reference  0.43 (-0.39; 1.25)  0.13 (-0.69; 0.95)  0.78 (-0.05; 1.61)  0.11 | Reference  0.06 (-1.51; 1.63)  -0.61 (-2.18; 0.96)  0.67 (-0.92; 2.27)  0.41 | Reference  0.12 (-0.39; 0.62)  -0.11 ( -0.62; 0.39)  0.14 (-0.37; 0.65)  0.83 | Reference  - 0.34 (-1.37; 0.68)  -0.54 (-1.57; 0.49)  -0.28 (-1.33; 0.76)  0.71 | Reference  0.19 (-0.16; 0.54)  0.07 (-0.28; 0.42)  0.20 (-0.15; 0.56) 0.49 | Reference  0.01 (-0.84; 0.86)  0.20 (-0.65; 1.05)  0.42 (-0.45; 1.29)  0.19 |
| **Parity continuous** | 0.14 (-0.03; 0.30) | 0.22 (-0.11; 0.55) | 0.05 ( -0.06; 0.15) | -0.003 (-0.22; 0.21) | 0.005 ( -0.07; 0.08) | 0.10 (-0.07; 0.27) |
| **Age at 1^st^ birth**  **<25**  **25-29**  **≥30**  **p-trend** | Reference  -0.40 (-0.87; 0.07)  -0.66 (-1.46; 0.13)  0.05 | Reference  -0.93 (-1.86; 0.01)  -0.31 (-1.91; 1.29)  0.32 | Reference  0.13 (-0.17; 0.43)  0.33 (-0.18; 0.83) 0.05 | Reference  0.08 (-0.52; 0.68)  0.76 (-0.28; 1.80)  0.19 | Reference  -0.09 ( -0.30; 0.12)  -0.19 ( -0.54; 0.16)  0.23 | Reference  -0.44 (-0.95; 0.06)  -0.50 (-1.36; 0.35)  0.12 |
| **Age at 1^st^ birth (per 5 years)** | -0.28 (-0.59; 0.03) | -0.30 (-0.92; 0.31) | 0.14 (-0.06; 0.34) | 0.24 (-0.16; 0.65) | -0.13 ( -0.26; 0.01) | -0.33 ( -0.66; 0.003) |
| **Age at menarche**  **<12**  **12**  **13**  **>13**  **p-trend** | -0.59 (-1.26; 0.07)  -0.80 (-1.43; -0.16)  -0.48 (-1.10; 0.13)  ref  0.06 | -1.36 (-2.67; -0.05)  -1.55 (-2.78; -0.32  -1.26 ( -2.47; -0.05)  ref  0.05 | 0.14 (-0.27; 0.55)  -0.12 (-0.50; 0.27)  0.14 (-0.23; 0.51)  ref  0.90 | 0.83 (-0.04; 1.70)  0.61 (-0.20; 1.43)  0.63 (-0.17; 1.44)  ref  0.09 | -0.28 (-0.57; 0.02)  -0.12 (-0.40; 0.15)  -0.10 (-0.36; 0.17)  Ref  0.07 | -0.53 (-1.22; 0.17)  -0.23 (-0.88; 0.43)  -0.71 (-1.35;-0.07)  Ref  0.46 |
| **Age at menarche (per 5 years)** | 0.63 (-0.17, 1.42) | 1.32 (-0.25, 2.89) | -0.09 (-0.58;0.40) | -0.92 (-1.97; 0.13) | 0.18 (-0.17; 0.53) | 0.11 (-0.73; 0.96) |
| **Time between menarche and age at 1^st^ birth (per 5 years)** | -0.36 (-0.66; -0.07) | -0.51 (-1.10; 0.08) | 0.14 (-0.05; 0.34) | 0.35 (-0.04; 0.74) | -0.14 (-0.27;-0.01) | -0.32 (-0.64; -0.004) |
| **Time since last birth (per 5 years)** | -0.05 (-0.30; 0.19) | -0.05 (-0.56; 0.45) | -0.03 (-0.20; 0.13) | -0.34 (-0.66; -0.02) | 0.00 ( -0.11; 0.12) | -0.12 (-0.39; 0.16) |

**Supplemental table 3. Associations of reproductive factors with log-transformed expression of stem cell markers in benign breast biopsy samples (β coefficients and 95% Confidence intervals) in mutually-adjusted models among parous women** ^a^

| **Reproductive factor** | **CD44** | | | | **CD24** | | | | | **ALDH1A1** | | | | |
| --- | --- | --- | --- | --- | --- | --- | --- | --- | --- | --- | --- | --- | --- | --- |
|  | **N** | **In Epithelium** | **N** | **In Stroma** | **N** | **In Epithelium** | **N** | **In Stroma** | **N** | | **In Epithelium** | **N** | **In Stroma** |  |
| **Breastfeeding, months**  **0-<1**  **1-<12**  **12-<24**  **≥24**  **p-trend** | 110  104  60  43  317 | Reference  0.22 (-0.32; 0.75)  -0.11 (-0.76; 0.54)  0.03 (-0.71; 0.76)  0.79 | 120  111  61  44  336 | Reference  -0.14 (-1.21; 0.94)  -0.23 (-1.57; 1.11)  -0.44 (-1.95; 1.07)  0.57 | 113  106  61  44  324 | Reference  0.14 (-0.22; 0.50)  -0.38 (-0.82; 0.06)  -0.09 (-0.58; 0.40)  0.26 | 124  113  62  45  344 | Reference  0.52 (-0.18; 1.22)  -0.20 (-1.06; 0.67)  -0.38 (-1.36; 0.59)  0.24 | 110  104  61  44  319 | | Reference  -0.08 (-0.33; 0.16)  -0.09 (-0.38; 0.21)  -0.02 (-0.35; 0.31)  0.88 | 116  109  62  40  327 | Reference  0.03 (-0.53; 0.60)  -0.15 (-0.84; 0.55)  -0.05 (-0.86; 0.76)  0.77 |  |
| **Parity**  **1**  **2**  **3**  **≥4**  **p-trend** | 30  91  104  92  317 | Reference  -0.13 (-1.01; 0.75)  -0.70 (-1.66; 0.27)  -0.25 (-1.34; 0.84)  0.53 | 33  99  110  94  336 | Reference  -0.48 (-2.26; 1.30)  -1.06 (-3.01; 0.88)  -0.14 (-2.35; 2.08)  0.98 | 31  96  101  96  324 | Reference  0.25 (-0.34; 0.83)  0.20 (-0.44; 0.85)  0.37 (-0.34; 0.83)  0.41 | 34  103  109  98  344 | Reference  -0.35 (-1.49; 0.79)  -0.72 (-1.97; 0.54)  -0.98 (-2.40; 0.43)  0.13 | 31  94  103  91  319 | | Reference  0.15 (-0.25; 0.54)  -0.06 (-0.50; 0.37)  0.04 (-0.45; 0.53)  0.69 | 31  98  105  93  327 | Reference  0.11 (-0.85; 1.06)  0.00 (-1.04; 1.05)  -0.14 (-1.33; 1.04)  0.63 |  |
| **Age at 1^st^ birth**  **<25**  **25-29**  **≥30**  **p-trend** | 176  116  25  317 | Reference  -0.45( -0.99; 0.08)  -1.15 (-2.21; -0.10)  0.02 | 189  121  26  336 | Reference  -0.85 (-1.93; 0.23)  -1.31 (-3.48; 0.86)  0.14 | 180  118  26  324 | Reference  0.28 (-0.07; 0.64)  0.41 (-0.29; 1.11)  0.15 | 190  127  27  344 | Reference  -0.22 (-0.91; 0.48)  -0.06 (-1.46; 1.32)  0.79 | 176  117  26  319 | | Reference  -0.07 (-0.31; 0.17)  -0.06 (-0.54; 0.42)  0.70 | 184  117  26  327 | Reference  -0.80 (-1.38; -0.22)  -0.84 (-1.98; 0.31)  0.04 |  |
| **Age at menarche**  **<12**  **12**  **13**  **>13**  **p-trend** | 78  76  96  67  317 | -0.95 (-1.64; -0.25)  -0.84 (-1.50; -0.18)  -0.65 (-1.28; -0.01)  Reference  0.01 | 79  85  100  72  336 | -2.03 (-3.44; -0.61)  -1.86 (-3.18; -0.55)  -1.78 (-3.07; -0.49)  Reference  0.01 | 76  76  100  72  324 | 0.13 (-0.34; 0.60)  -0.17 (-0.61; 0.27)  0.19 (-0.22; 0.61)  Reference  0.90 | 80  86  101  77  344 | 0.72 (-0.20; 1.64)  0.65 (-0.19; 1.50)  0.50 (-0.33; 1.33)  Reference  0.12 | 74  75  100  70  319 | | -0.27 (-0.58; 0.05)  -0.22 (-0.51; 0.08)  -0.06 (-0.34; 0.22)  Reference  0.05 | 78  81  98  70  327 | -0.70 (-1.45; 0.05)  -0.54 (-1.24; 0.16)  -0.87 (-1.55; -0.20)  Reference  0.20 |  |
|  | 317 | -0.20 (-0.55; 0.15) | 336 | -0.28 (-0.98; 0.43) | 324 | 0.07 (-0.16; 0.30) | 344 | -0.54 (-1.00; -0.08) | 319 | | -0.00 (-0.16; 0.16) | 327 | -0.22 (-0.60; 0.15) |  |

^a^ The mutually-adjusted model includes age (continuous), BMI (continuous), age at menarche, parity, age at first child’s birth, breastfeeding, time since last birth, a family history of breast cancer (Yes/No), menopausal status/postmenopausal hormone use (premenopausal, postmenopausal/no hormones, postmenopausal/past hormones, postmenopausal/current hormones, postmenopausal/unknown hormone use status), NHS cohort (NHSI, NHSII), benign breast disease subtype (non-proliferative, proliferative without atypia, proliferative with atypia), and alcohol use (none, >0-<5, ≥5 g/day)

**Supplementary table 4. Association of reproductive factors with dichotomous expression of stem cell markers (Odds ratios [OR] and 95% Confidence intervals [CI]), using 10% cut-off for staining positivity**

| **Reproductive factor** | **CD44** | | | | **CD24** | | | | **ALDH1A1** | | | |
| --- | --- | --- | --- | --- | --- | --- | --- | --- | --- | --- | --- | --- |
|  | **N** | **In Epithelium** | **N** | **In Stroma** | **N** | **In Epithelium** | **N** | **In Stroma** | **N** | **In Epithelium** | **N** | **In Stroma** |
| **Nulliparity^a^**  **Nulliparous**  **Parous** | 28  335 | 1.16 (0.46; 2.91)  Ref | 34  354 | 1.36 (0.60; 3.12)  Ref | 34  342 | 0.97 (0.39; 2.44)  Ref | 36  362 | 0.95 (0.39; 2.33)  Ref | 29  336 | 4.65 (1.26; 17.24)  Ref | 30  345 | 1.45 (0.63; 3.36)  Ref |
| **Breastfeeding, months^b^**  **0-<1**  **1-<12**  **12-<24**  **≥24**  **p-trend** | 110  105  63  45  323 | Ref  1.40 (0.73; 2.38)  0.71 (0.34; 1.50)  0.78 (0.34; 1.82)  0.26 | 120  112  64  46  342 | Ref  0.85 (0.47; 1.52)  0.57 (0.27; 1.21)  0.67 (0.28; 1.56)  0.20 | 113  107  64  46  330 | Ref  1.32 (0.63; 2.77)  0.91 (0.38; 2.17)  0.90 (0.35; 2.31)  0.62 | 124  114  65  47  350 | Ref  1.74 (0.93; 3.26)  0.70 (0.31; 1.58)  0.42 (0.15; 1.20)  0.03 | 110  105  64  46  325 | Ref  0.99 (0.49; 2.01)  0.63 (0.29; 1.41)  0.89 (0.36; 2.23)  0.54 | 116  110  65  42  333 | Ref  1.02 (0.58; 1.81)  1.10 (0.55; 2.19)  1.03 (0.46; 2.35)  0.88 |
| **Parity ^c^**  **1**  **2**  **3**  **≥4**  **p-trend** | 34  96  105  97  332 | Ref  0.78 (0.31; 1.92)  0.66 (0.26; 1.71)  1.25 (0.46; 3.37)  0.41 | 37  104  111  99  351 | Ref  0.57 (0.23; 1.43)  0.48 (0.19; 1.24)  1.00 (0.38; 2.59)  0.34 | 35  101  102  101  339 | Ref  1.46 (0.51; 4.18)  1.09 (0.38; 3.13)  1.40 (0.47; 4.16)  0.83 | 38  108  110  103  359 | Ref  0.63 (0.23; 1.69)  0.55 (0.20; 1.53)  0.61 (0.21; 1.75)  0.53 | 35  99  104  96  334 | Ref  1.20 (0.47; 3.09)  1.01 (0.38; 2.66)  1.57 (0.56; 4.41)  0.45 | 35  103  106  98  342 | Ref  1.12 (0.46; 2.76)  1.21 (0.48; 3.07)  1.16 (0.44; 3.00)  0.80 |
| **Parity continuous ^c^** | 332 | 1.05 (0.85; 1.28) | 351 | 1.14 (0.95; 1.38) | 339 | 1.12 (0.88; 1.42) | 359 | 0.92 (0.75; 1.13) | 334 | 1.04 (0.83; 1.30) | 342 | 0.92 (0.76; 1.11) |
| **Age at 1^st^ birth ^d^**  **<25**  **25-29**  **≥30**  **p-trend** | 183  124  29  332 | Ref  0.81 (0.47; 1.41)  0.66 (0.26; 1.67)  0.32 | 193  128  30  351 | Ref  1.16 (0.69; 1.95)  1.16 (0.46; 2.94)  0.63 | 184  125  30  339 | Ref  1.05 (0.57; 1.91)  5.51 (1.12; 27.18)  0.05 | 194  134  31  359 | Ref  0.80 (0.45; 1.43)  0.69 (0.25; 1.97)  0.39 | 180  124  30  334 | Ref  0.89 (0.49; 1.60)  0.62 (0.24; 1.62)  0.33 | 188  124  30  342 | Ref  0.74 (0.44; 1.24)  0.99 (0.40; 2.41)  0.65 |
| **Age at 1^st^ birth, (per 5 years) ^d^** | 332 | 0.76 (0.53; 1.09) | 351 | 1.18 (0.82; 1.69) | 339 | 1.59 (1.03; 2.48) | 359 | 0.83 (0.56; 1.25) | 334 | 0.73 (0.50; 1.06) | 342 | 0.80 (0.57; 1.14) |
| **Age at menarche ^e^**  **<12**  **12**  **13**  **>13**  **p-trend** | 88  95  109  71  363 | 0.52 (0.23; 1.18)  0.36 (0.16; 0.78)  0.51 (0.24; 1.10)  Ref  0.12 | 91  106  114  77  388 | 0.68 (0.34; 1.35)  0.64 (0.34; 1.21)  0.64 (0.34; 1.20)  Ref  0.31 | 87  98  114  77  376 | 1.04 (0.45; 2.39)  0.89 (0.40; 1.96)  1.15 (0.53; 2.48)  Ref  0.89 | 91  108  116  83  398 | 1.77 (0.78; 3.99)  1.65 (0.77; 3.52)  2.17 (1.04; 4.53)  Ref  0.35 | 83  93  114  75  365 | 0.47 (0.20; 1.11)  0.56 (0.25; 1.28)  0.69 (0.31; 1.53)  Ref  0.07 | 86  102  112  75  375 | 1.13 (0.56; 2.26)  0.79 (0.41; 1.51)  0.80 (0.42; 1.51)  Ref  0.72 |
| **Age at menarche, (per 5 years) ^e^** | 363 | 1.79 (0.72; 4.45) | 388 | 1.54 (0.67; 3.56) | 376 | 0.86 (0.32; 2.32) | 398 | 0.72 (0.28; 1.84) | 365 | 2.28 (0.85; 6.10) | 375 | 0.86 (0.37; 2.01) |
| **Time between menarche and age at 1^st^ birth, (per 5 years) ^f^** | 332 | 0.72 (0.51; 1.01) | 351 | 1.04 (0.74; 1.46) | 339 | 1.55 (1.02; 2.35) | 359 | 0.87 (0.59; 1.27) | 334 | 0.68 (0.47; 0.98) | 342 | 0.80 (0.58; 1.12) |
| **Time since last birth (per 5 years)^b^** | 325 | 0.70 (0.43; 1.15) | 351 | 1.07 (0.69; 1.67) | 332 | 1.19 (0.71; 1.98) | 352 | 1.85 (1.05; 3.26) | 327 | 0.60 (0.33; 1.07) | 335 | 0.60 ( 0.38; 0.94) |

^a^ Adjusted for age (continuous), BMI (continuous), age at menarche (<12, 12, 13, >13), a family history of breast cancer (Yes/No), menopausal status/postmenopausal hormone use (premenopausal, postmenopausal/no hormones, postmenopausal/past hormones, postmenopausal/current hormones, postmenopausal/unknown hormone use status), NHS cohort (NHSI, NHSII), benign breast disease subtype (non-proliferative, proliferative without atypia, proliferative with atypia),and alcohol use (none, >0-<5, ≥5 g/day)

**^b^** Among parous women only: adjusted for age (continuous), BMI (continuous), age at menarche (<12, 12, 13, >13), parity, age at first child’s birth, a family history of breast cancer (Yes/No), menopausal status/postmenopausal hormone use (premenopausal, postmenopausal/no hormones, postmenopausal/past hormones, postmenopausal/current hormones, postmenopausal/unknown hormone use status), NHS cohort (NHSI, NHSII), benign breast disease subtype (non-proliferative, proliferative without atypia, proliferative with atypia), and alcohol use (none, >0-<5, ≥5 g/day)

**^c^** Among parous women only: adjusted for age (continuous), BMI (continuous), age at first birth, age at menarche (<12, 12, 13, >13), a family history of breast cancer (Yes/No), menopausal status/postmenopausal hormone use (premenopausal, postmenopausal/no hormones, postmenopausal/past hormones, postmenopausal/current hormones, postmenopausal/unknown hormone use status), NHS cohort (NHSI, NHSII), benign breast disease subtype (non-proliferative, proliferative without atypia, proliferative with atypia), and alcohol use (none, >0-<5, ≥5 g/day)

**^d^** Among parous women only: adjusted for age (continuous), BMI (continuous), parity, age at menarche (<12, 12, 13, >13), a family history of breast cancer (Yes/No), menopausal status/postmenopausal hormone use (premenopausal, postmenopausal/no hormones, postmenopausal/past hormones, postmenopausal/current hormones, postmenopausal/unknown hormone use status), NHS cohort (NHSI, NHSII), benign breast disease subtype (non-proliferative, proliferative without atypia, proliferative with atypia), and alcohol use (none, >0-<5, ≥5 g/day)

**^e^** Adjusted for age (continuous), BMI (continuous), parous status (nulliparous, parous), a family history of breast cancer (Yes/No), menopausal status/postmenopausal hormone use (premenopausal, postmenopausal/no hormones, postmenopausal/past hormones, postmenopausal/current hormones, postmenopausal/unknown hormone use status), NHS cohort (NHSI, NHSII), benign breast disease subtype (non-proliferative, proliferative without atypia, proliferative with atypia), and alcohol use (none, >0-<5, ≥5 g/day)

**^f^** Among parous women only: adjusted for age (continuous), BMI (continuous), parity, a family history of breast cancer (Yes/No), menopausal status/postmenopausal hormone use (premenopausal, postmenopausal/no hormones, postmenopausal/past hormones, postmenopausal/current hormones, postmenopausal/unknown hormone use status), NHS cohort (NHSI, NHSII), benign breast disease subtype (non-proliferative, proliferative without atypia, proliferative with atypia), and alcohol use (none, >0-<5, ≥5 g/day)

**Supplementary table 5.** **Associations of reproductive factors with log-transformed expression of stem cell markers in benign breast biopsy samples (β coefficients and 95% Confidence intervals) in premenopausal women**

| **Reproductive factor** | **CD44** | | | | **CD24** | | | | **ALDH1A1** | | | |
| --- | --- | --- | --- | --- | --- | --- | --- | --- | --- | --- | --- | --- |
|  | **N** | **In Epithelium** | **N** | **In Stroma** | **N** | **In Epithelium** | **N** | **In Stroma** | **N** | **In Epithelium** | **N** | **In Stroma** |
| **Nulliparity^a^**  **Nulliparous**  **Parous** | 20  251 | 0.67 (-0.40; 1.74)  Reference | 24  263 | 0.32 (-1.47; 2.11)  Reference | 24  253 | 0.27 (-0.33; 0.87)  Reference | 25  264 | -0.31 (-1.43; 0.81)  Reference | 21  251 | 0.41 (-0.01; 0.83)  Reference | 22  256 | 0.75 (-0.23; 1.74)  Reference |
| **Breastfeeding, months^b^**  **0-<1**  **1-<12**  **12-<24**  **≥24**  **p-trend** | 79  73  49  40  241 | Reference  0.16 (-0.55; 0.87)  -0.27 (-1.11; 0.57)  0.16 (-0.73; 1.06)  0.99 | 86  76  50  41  253 | Reference  -0.59 (-1.86; 0.68)  -0.28 (-1.78; 1.23)  -0.44 (-2.07; 1.18)  0.75 | 80  73  51  39  243 | Reference   - 1. (-1.46; 0.47)   -0.52 (-1.06; 0.02)  -0.09 (-0.68; 0.50)  0.36 | 87  76  51  40  254 | Reference  0.17 (-0.64; 0.98)  -0.01 (-0.96; 0.94)  -0.01 (-1.05; 1.03)  0.88 | 80  71  51  40  242 | Reference  0.01 (-0.29; 0.32)  0.002 (-0.35; 0.35)  -0.02 (-0.40; 0.36)  0.90 | 86  72  51  37  246 | Reference  0.16 (-0.57; 0.88)  -0.12 (-0.95; 0.72)  0.19 (-0.76; 1.14)  0.90 |
| **Parity ^c^**  **1**  **2**  **3**  **≥4**  **p-trend** | 28  81  74  65  248 | Reference  0.70 (-0.30; 1.70)  0.07 (-0.97; 1.11)  0.55 (-0.53; 1.63)  0.89 | 31  86  76  67  260 | Reference  0.56 (-0.12; 2.32)  0.03 (-1.83; 1.88)  0.08 (-1.86; 2.02)  0.69 | 29  85  71  65  250 | Reference  0.29 (-0.35; 0.93)  0.16 (-0.50; 0.83)  0.30 (-0.39; 0.99)  0.65 | 32  87  76  66  261 | Reference  -0.34 (-1.43; 0.75)  -0.26 (-1.40; 0.89)  -0.37 (-1.57; 0.83)  0.69 | 29  83  72  65  249 | Reference  0.31 (-0.10; 0.73)  0.11 (-0.32; 0.55)  0.20 (-0.25; 0.65)  0.99 | 29  86  74  64  253 | Reference  0.11 (-0.89; 1.11)  0.46 (-0.60; 1.52)  0.53 (-0.57; 1.63)  0.20 |
| **Parity continuous ^c^** | 248 | 0.01 (-0.22; 0.24) | 260 | -0.08 (-0.50; 0.33) | 250 | 0.08 (-0.06; 0.23) | 261 | -0.02 (-0.28; 0.24) | 249 | -0.07 (-0.16; 0.03) | 253 | -0.002 (-0.23; 0.23) |
| **Age at 1^st^ birth ^d^**  **<25**  **25-29**  **≥30**  **p-trend** | 136  87  25  248 | Reference  -0.37 (-0.98; 0.25)  -0.82 (-1.83; 0.19)  0.08 | 143  9  26  260 | Reference  -0.81 (-1.92; 0.30)  -1.10 (-2.93; 0.73)  0.14 | 137  88  25  250 | Reference  0.33 (-0.06; 0.73)  0.53 (-0.12; 0.18)  0.06 | 142  93  26  261 | Reference  0.44 (-0.25; 1.12)  0.81 (-0.34; 1.95)  0.11 | 135  88  26  249 | Reference  -0.14 (-0.40; 0.12)  -0.27 (-0.69; 0.16)  0.16 | 139  88  26  253 | Reference  -0.93 (-1.55; -0.31)  -0.63 (-1.64; 0.38)  0.05 |
| **Age at 1^st^ birth (per 5 years) ^d^** | 248 | -0.39 (-0.80; 0.02) | 260 | -0.66 (-1.38; 0.06) | 250 | 0.31 (0.04; 0.57) | 261 | 0.34 (-0.11; 0.80) | 249 | -0.25 (-0.41; -0.08) | 253 | -0.54 (-0.95; -0.13) |
| **Age at menarche ^e^**  **<12**  **12**  **13**  **>13**  **p-trend** | 73  64  89  45  271 | -0.48 (-1.37; 0.41)  -0.97 (-1.85; -0.09)  -0.59 (-1.41; 0.23)  ref  0.33 | 74  72  93  48  287 | -0.64 (-2.21; 0.93)  -1.41 (-2.95; 0.12)  -1.06 (-2.51; 0.38)  ref  0.54 | 71  65  92  49  277 | 0.22 (-0.32; 0.75)  -0.26 (-0.79; 0.27)  0.15 (-0.34; 0.64)  Ref  0.78 | 73  72  93  51  289 | 1.31 (0.31; 2.31)  0.59 (-0.38; 1.57)  0.26 (-0.65; 1.18)  Ref  0.01 | 68  63  92  49  272 | -0.12 (-0.47; 0.24)  -0.13 (-0.48; 0.22)  0.01 (-0.32; 0.33)  Ref  0.37 | 72  71  89  46  278 | -0.69 (-1.53; 0.16)  -0.70 (-1.53; 0.13)  -0.75 (-1.53; 0.04)  Ref  0.23 |
| **Age at menarche (per 5 years) ^e^** | 271 | 0.46 (-0.56; 1.49) | 287 | 0.52 (-1.29; 2.33) | 277 | -0.07 (-0.70; 0.55) | 289 | -1.43 (-2.59; -0.27) | 272 | 0.11 (-0.31; 0.52) | 278 | 0.53 (-0.46; 1.51) |
| **Time between menarche and age at 1^st^ birth (per 5 years) ^f^** | 248 | -0.47 (-0.85; -0.09) | 260 | -0.84 (-1.53; -0.16) | 250 | 0.24 (-0.01; 0.49) | 261 | 0.38 (-0.05; 0.81) | 249 | -0.23 (-0.39; -0.07) | 253 | -0.59 (-0.98; -0.21) |
| **Time since last birth (per 5 years)^b^** | 241 | -0.37 (-0.89; 0.15) | 253 | -0.67 (-1.63; 0.29) | 243 | 0.22 (-0.13; 0.56) | 254 | -0.43 (-1.04; 0.17) | 242 | -0.14 (-0.37; 0.09) | 246 | -0.60 (-1.17; -0.03) |

^a^ Adjusted for age (continuous), BMI (continuous), age at menarche (<12, 12, 13, >13), a family history of breast cancer (Yes/No), NHS cohort (NHSI, NHSII), benign breast disease subtype (non-proliferative, proliferative without atypia, proliferative with atypia),and alcohol use (none, >0-<5, ≥5 g/day)

**^b^** Among parous women only: adjusted for age (continuous), BMI (continuous), age at menarche (<12, 12, 13, >13), parity, age at first child’s birth, a family history of breast cancer (Yes/No), NHS cohort (NHSI, NHSII), benign breast disease subtype (non-proliferative, proliferative without atypia, proliferative with atypia), and alcohol use (none, >0-<5, ≥5 g/day)

**^c^** Among parous women only: adjusted for age (continuous), BMI (continuous), age at first birth, age at menarche (<12, 12, 13, >13), a family history of breast cancer (Yes/No), NHS cohort (NHSI, NHSII), benign breast disease subtype (non-proliferative, proliferative without atypia, proliferative with atypia), and alcohol use (none, >0-<5, ≥5 g/day)

**^d^** Among parous women only: adjusted for age (continuous), BMI (continuous), parity, age at menarche (<12, 12, 13, >13), a family history of breast cancer (Yes/No), NHS cohort (NHSI, NHSII), benign breast disease subtype (non-proliferative, proliferative without atypia, proliferative with atypia), and alcohol use (none, >0-<5, ≥5 g/day)

**^e^** Adjusted for age (continuous), BMI (continuous), parous status (nulliparous, parous), a family history of breast cancer (Yes/No), NHS cohort (NHSI, NHSII), benign breast disease subtype (non-proliferative, proliferative without atypia, proliferative with atypia), and alcohol use (none, >0-<5, ≥5 g/day)

**^f^** Among parous women only: adjusted for age (continuous), BMI (continuous), parity, a family history of breast cancer (Yes/No), NHS cohort (NHSI, NHSII), benign breast disease subtype (non-proliferative, proliferative without atypia, proliferative with atypia), and alcohol use (none, >0-<5, ≥5 g/day)

**Supplementary table 6.** **Associations of reproductive factors with log-transformed expression of stem cell markers in benign breast biopsy samples (β coefficients and 95% Confidence intervals) in postmenopausal women**

| **Reproductive factor** | **CD44** | | | | **CD24** | | | | **ALDH1A1** | | | |
| --- | --- | --- | --- | --- | --- | --- | --- | --- | --- | --- | --- | --- |
|  | **N** | **In Epithelium** | **N** | **In Stroma** | **N** | **In Epithelium** | **N** | **In Stroma** | **N** | **In Epithelium** | **N** | **In Stroma** |
| **Nulliparity^a^**  **Nulliparous**  **Parous** | 61  6 | -0.05 (-1.16; 1.06)  Reference | 67  8 | 2.00 (-1.06; 5.06)  Reference | 8  65 | -0.14 (-0.87; 0.58)  Reference | 9  74 | -0.31 (-2.70; 2.08)  Reference | 6  61 | -0.42 (-0.98; 0.15)  Reference | 6  66 | 0.07 (-1.29; 1.44)  Reference |
| **Breastfeeding, months^b^**  **0-<1**  **1-<12**  **12-<24**  **≥24**  **p-trend** | 27  23  7  4  61 | Reference  0.72 (0.08; 1.35)  0.47 (-0.49; 1.44)  0.61 (-0.56; 1.78)  0.28 | 30  26  7  4  67 | Reference  2.24 (0.28; 4.20)  0.92 (-2.09; 3.94)  3.07 (-0.72; 6.85)  0.16 | 28  25  6  6  65 | Reference  0.34 (-0.18; 0.87)  0.30 (-0.57; 1.17)  -0.02 (-0.89; 0.85)  0.98 | 32  29  7  6  74 | Reference  0.94 (-0.77; 2.65)  -0.86 (-3.59; 1.87)  -0.89 (-3.80; 2.02)  0.35 | 26  24  6  5  61 | Reference  -0.26 (-0.62; 0.11)  -0.47 (-1.05; 0.11)  -0.17 (-0.82; 0.48)  0.39 | 26  29  7  4  66 | Reference  -0.30 (-1.15; 0.55)  -0.67 (-2.01; 0.67)  -2.24 (-3.96; -0.51)  0.01 |
| **Parity ^c^**  **1**  **2**  **3**  **≥4**  **p-trend** | 3  13  21  24  61 | Reference  -2.37 (-4.23; -0.51)  -1.56 (-3.51; 0.39)  -0.75 (-2.69; 1.18)  <0.01 | 3  16  24  24  67 | Reference  -6.13 (-12.19; -0.08)  -5.66 (-11.94; 0.63)  -2.53 (-8.83; 3.78)  0.01 | 3  13  21  28  65 | Reference  0.24 (-1.26; 1.75)  0.32 (-1.21; 1.85)  0.68 (-0.87; 2.22)  0.13 | 3  18  24  29  74 | Reference  1.32 (-4.00; 6.63)  1.05 (-4.37; 6.47)  1.96 (-3.54; 7.46)  0.40 | 3  14  21  23  61 | Reference  -0.62 (-1.63; 0.39)  -0.55 (-1.59; 0.49)  -0.39 (-1.43; 0.65)  0.50 | 3  15  22  26  66 | Reference  0.45 (-2.16; .06)  -0.68 (-3.39; 2.04)  -0.49 (-3.16; 2.19)  0.15 |
| **Parity continuous ^c^** | 61 | 0.19 (-0.07; 0.45) | 67 | 0.66 (-0.08; 1.40) | 65 | 0.07 (-0.11; 0.26) | 74 | 0.41 (-0.16; 0.98) | 61 | 0.01 (-0.12; 0.14) | 66 | -0.16 (-0.45; 0.13) |
| **Age at 1^st^ birth ^d^**  **<25**  **25-29**  **≥30**  **p-trend** | 30  29  2  61 | Reference  -0.11 (-0.75; 0.53)  0.71 (-1.05; 2.47)  0.73 | 35  30  2  67 | Reference  0.45 (-1.48; 2.38)  -2.53 (-8.83; 3.78)  0.30 | 33  30  2  65 | Reference  0.26 (-0.22; 0.75)  0.20 (-1.21; 1.61)  0.42 | 38  34  2  74 | Reference  -0.24 (-1.84; 1.35)  0.92 (-3.98; 5.82)  0.97 | 30  29  2  61 | Reference  0.09 (-0.25; 0.43)  -0.49 (-1.42; 0.45)  0.69 | 34  30  2  66 | Reference  0.59 (-0.26; 1.44)  -0.12 (-2.47; 2.23)  0.48 |
| **Age at 1^st^ birth (per 5 years) ^d^** | 61 | -0.15 (-0.83; 0.52) | 67 | 0.90 (-1.05; 2.86) | 65 | 0.01 (-0.45; 0.48) | 74 | 0.20 (-1.37; 1.76) | 61 | 0.05 (-0.28; 0.37) | 66 | 0.28 (-0.54; 1.09) |
| **Age at menarche ^e^**  **<12**  **12**  **13**  **>13**  **p-trend** | 8  23  18  18  67 | -2.38 (-3.49; -1.27)  -0.85 (-1.78; 0.08)  -1.21 (-2.12; -0.29)  Ref  <0.001 | 10  26  18  21  75 | -5.72 (-8.93; -2.50)  -1.66 (-4.15; 0.83)  -3.01 (-5.75; -0.27)  Ref  0.01 | 9  24  20  20  73 | -0.73 (-1.51; 0.06)  -0.46 (-1.09; 0.17)  0.02 (-0.59; 0.63)  Ref  0.03 | 11  27  21  24  83 | -1.26 (-3.70; 1.19)  0.31 (-1.56; 2.19)  1.77 (-0.17; 3.72)  Ref  0.34 | 8  22  19  18  67 | -0.91 (-1.50; -0.33)  -0.67 (-1.15; -0.18)  -0.15 (-0.61; 0.32)  Ref  <0.001 | 7  23  20  22  72 | -0.18 (-1.56; 1.20)  -0.56 (-1.59; 0.47)  -1.21 (-2.23; -0.19)  Ref  0.84 |
| **Age at menarche (per 5 years) ^e^** | 67 | 2.37 (0.81; 3.93) | 75 | 5.64 (2.11; 10.07) | 73 | 1.18 (0.14; 2.22) | 83 | 2.02 (-1.28; 5.32) | 67 | 1.39 (0.62; 2.16) | 72 | 0.49 (-1.30; 2.27) |
| **Time between menarche and age at 1^st^ birth (per 5 years) ^f^** | 61 | -0.26 (-1.02; 0.49) | 67 | 0.43 (-1.66; 2.52) | 65 | -0.05 (-0.54; 0.44) | 74 | 0.05 (-1.60; 1.69) | 61 | -0.0001 (-0.38; 0.38) | 66 | 0.20 (-0.65; 1.06) |
| **Time since last birth (per 5 years)^b^** | 61 | -0.02 (-0.61; 0.58) | 67 | 0.51 (-1.23; 2.25) | 65 | 0.00 (-0.39; 0.40) | 74 | -1.20 (-2.49; 0.09) | 61 | -0.42 (-0.70; -0.14) | 66 | -0.12 (-0.83; 0.59) |

^a^ Adjusted for age (continuous), BMI (continuous), age at menarche (<12, 12, 13, >13), a family history of breast cancer (Yes/No), postmenopausal hormone use (no hormones, past hormones, current hormones, unknown hormone use status), NHS cohort (NHSI, NHSII), benign breast disease subtype (non-proliferative, proliferative without atypia, proliferative with atypia),and alcohol use (none, >0-<5, ≥5 g/day)

**^b^** Among parous women only: adjusted for age (continuous), BMI (continuous), age at menarche (<12, 12, 13, >13), parity, age at first child’s birth, a family history of breast cancer (Yes/No), postmenopausal hormone use (no hormones, past hormones, current hormones, unknown hormone use status), NHS cohort (NHSI, NHSII), benign breast disease subtype (non-proliferative, proliferative without atypia, proliferative with atypia), and alcohol use (none, >0-<5, ≥5 g/day)

**^c^** Among parous women only: adjusted for age (continuous), BMI (continuous), age at first birth, age at menarche (<12, 12, 13, >13), a family history of breast cancer (Yes/No), postmenopausal hormone use (no hormones, past hormones, current hormones, unknown hormone use status), NHS cohort (NHSI, NHSII), benign breast disease subtype (non-proliferative, proliferative without atypia, proliferative with atypia), and alcohol use (none, >0-<5, ≥5 g/day)

**^d^** Among parous women only: adjusted for age (continuous), BMI (continuous), parity, age at menarche (<12, 12, 13, >13), a family history of breast cancer (Yes/No), postmenopausal hormone use (no hormones, past hormones, current hormones, unknown hormone use status), NHS cohort (NHSI, NHSII), benign breast disease subtype (non-proliferative, proliferative without atypia, proliferative with atypia), and alcohol use (none, >0-<5, ≥5 g/day)

**^e^** Adjusted for age (continuous), BMI (continuous), parous status (nulliparous, parous), a family history of breast cancer (Yes/No), postmenopausal hormone use (no hormones, past hormones, current hormones, unknown hormone use status), NHS cohort (NHSI, NHSII), benign breast disease subtype (non-proliferative, proliferative without atypia, proliferative with atypia), and alcohol use (none, >0-<5, ≥5 g/day)

**^f^** Among parous women only: adjusted for age (continuous), BMI (continuous), parity, a family history of breast cancer (Yes/No), postmenopausal hormone use (no hormones, past hormones, current hormones, unknown hormone use status), NHS cohort (NHSI, NHSII), benign breast disease subtype (non-proliferative, proliferative without atypia, proliferative with atypia), and alcohol use (none, >0-<5, ≥5 g/day)
